# Pathways to Triplet or Singlet Oxygen during the Dissociation of Alkali Metal Superoxides: Insights by Multireference Calculations of Molecular Model Systems

**DOI:** 10.1002/chem.201904110

**Published:** 2020-01-21

**Authors:** Aleksandr Zaichenko, Daniel Schröder, Jürgen Janek, Doreen Mollenhauer

**Affiliations:** ^1^ Institute of Physical Chemistry Justus-Liebig University Giessen Heinrich-Buff-Ring 17 35392 Giessen Germany; ^2^ Center for Materials Research Justus-Liebig University Giessen Heinrich-Buff-Ring 16 35392 Giessen Germany

**Keywords:** ab initio calculations, lithium, oxygen, quantum chemistry, sodium

## Abstract

Recent experimental investigations demonstrated the generation of singlet oxygen during charging at high potentials in lithium/oxygen batteries. To contribute to the understanding of the underlying chemical reactions a key step in the mechanism of the charging process, namely, the dissociation of the intermediate lithium superoxide to oxygen and lithium, was investigated. Therefore, the corresponding dissociation paths of the molecular model system lithium superoxide (LiO_2_) were studied by CASSCF/CASPT2 calculations. The obtained results indicate the presence of different dissociation paths over crossing points of different electronic states, which lead either to the energetically preferred generation of triplet oxygen or the energetically higher lying formation of singlet oxygen. The dissociation to the corresponding superoxide anion is energetically less preferred. The understanding of the detailed reaction mechanism allows the design of strategies to avoid the formation of singlet oxygen and thus to potentially minimize the degradation of materials in alkali metal/oxygen batteries. The calculations demonstrate a qualitatively similar but energetically shifted behavior for the homologous alkali metals sodium and potassium and their superoxide species. Fundamental differences were found for the covalently bound hydroperoxyl radical.

## Graphical abstract

**Crossing paths**: To understand the underlying chemical reactions in lithium/oxygen batteries the dissociation of the intermediate lithium superoxide was investigated by multireference calculations of molecular model systems. The elucidated reaction mechanism involving two paths and two crossing points (CP) allows the design of strategies that avoid the formation of singlet oxygen and can be qualitatively transferred to the homologous alkali metals sodium and potassium.

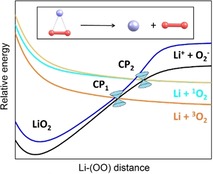

## Introduction

The electrochemical oxidation of alkali metals and subsequent reduction of the oxidation products attracts great interest due to the recent development of alkali metal/oxygen batteries.^1^, ^2^ Lithium‐, sodium‐, and potassium‐based metal/oxygen batteries are investigated as potential post‐lithium‐ion battery systems.^2^, ^3^ The Li/O_2_ system is especially appealing owing to its very high theoretical energy density, which is about ten times higher than that of the lithium‐ion battery.^1^

A nonaqueous alkali metal/oxygen battery comprises a solid metal anode, a porous separator filled with organic, liquid electrolyte, and a porous cathode that is flooded with the electrolyte. Oxygen is not carried inside the battery; it is provided by the surrounding gas atmosphere at the cathode. Two main processes occur at the cathode of an alkali metal/oxygen battery: 1) discharging, which proceeds with an oxygen reduction reaction (ORR), in which electrons, metal ions, and dissolved oxygen are converted to a solid metal oxide; and 2) charging, which is associated with the oxygen evolution reaction (OER), in which decomposition of the solid metal oxide releases oxygen species, metal ions, and electrons. The different alkali metals form different oxygen reduction compounds.^2^ Whereas in the case of the Li/O_2_ system lithium peroxide (Li_2_O_2_) is observed as product of the ORR, the superoxide phases NaO_2_ and KO_2_ are reported for the Na/O_2_ and K/O_2_ systems, respectively.^3^ Depending on the cell configuration and the electrolytes used, also the formation of Na_2_O_2_ (mostly as peroxide hydrate Na_2_O_2_ **⋅** *x* H_2_O) has been reported.^4^, ^5^, ^6^ Traces of water and protons in the electrolyte can alter the nature of the sodium oxide species formed.^7^, ^8^

Although the final product of the ORR in the Li/O_2_ battery is lithium peroxide, the importance of lithium superoxide as an intermediate has been demonstrated in experimental studies on ORR and OER.^9^, ^10^ On the basis of these studies, the first step of the OER process is described as the reaction from the lithium peroxide to the superoxide [Eq. (1)]:

(1) $$\mathrm{Li}{}_{2}O{}_{2}\to \mathrm{LiO}{}_{2}+\mathrm{Li}{}^{+}+e{}^{-}$$

Secondly, disproportionation of the superoxide can take place [Eq. (2)]:

(2) $$2\;\mathrm{LiO}{}_{2}\to \mathrm{Li}{}_{2}O{}_{2}+O{}_{2}{}^{\left[11\right]}$$

The third reaction corresponds to the dissociation of the superoxide intermediate to oxygen, a lithium ion, and an electron [Eq. (3)]:

(3) $$\mathrm{LiO}{}_{2}\to \mathrm{Li}{}^{+}+O{}_{2}+e{}^{-}$$

For the ORR process the same elementary reactions—except for the disproportionation—have been proposed to take place in the reverse direction.

Recently, experimental investigations have demonstrated that the generation of singlet oxygen at potentials higher than 3.5 V might play a role in the charging processes of Li/O_2_ systems.^12^ The highly reactive singlet oxygen has been detected as small fraction of 0.5 % of the produced oxygen species, whereas some part of the singlet oxygen might already have been quenched in side reactions with the cathode materials. Further, Mahne et al. found that singlet oxygen forms at the cathode from the onset of charging in a Li/O_2_ system,^13^ and it is presumed that the singlet oxygen can destroy the electrode and electrolytes in the battery cell. Mahne et al. even accounted for the main part of the side reactions to singlet oxygen generation.^13^ Thus, besides the generation of triplet oxygen, the generation of singlet oxygen needs to be understood in detail in order to develop strategies to avoid its formation and thus to diminish the degradation of the cathode materials. The formation of singlet oxygen has also been detected as the main driver for degradation in Na/O_2_ cells.^14^

The ORR and the OER in alkali metal/oxygen systems have already been investigated by several theoretical studies. Calculations of the bulk phase of reduced or oxidized compounds and surface as well as molecular or cluster structures have been reported. DFT calculations of the OER mechanism on different low‐index surfaces of lithium peroxide provide understanding of the formation superoxide intermediate as well as the dependence on the surface orientation.^15^ The energetically preferred OER path takes place via superoxide intermediates. Also, DFT calculations of the mixture of Li_2_O_2_/LiO_2_ as bulk phase were used to explain magnetic properties of the system.^16^ An insight into the LiO_2_ disproportionation reaction and its dependence on the cluster size and surface composition has been given by DFT calculations on small clusters.^17^ Also, the stability of LiO_2_ dimers in the gas phase was evaluated by coupled‐cluster with singles, doubles, and perturbative triples [CCSD(T)] calculations, which indicated low barriers for the disproportionation to Li_2_O_2_ and the triplet oxygen molecule.^18^ On the basis of these studies Mahne et al. identified disproportionation as a possible source of singlet oxygen.^13^

Furthermore, the stability of different lithium peroxide and sodium peroxide clusters as well as superoxide clusters has been determined by means of the surface energy of the corresponding oxides.^19^ Also, molecular lithium and sodium superoxides containing just one alkali metal atom have been investigated. These model systems have the advantage that a higher level of theory could be used for their description, namely Møller–Plesset perturbation theory of the fourth order (MP4), CCSD(T), and complete active space self‐consistent (CASSCF) calculations with an active space smaller than (7,8). These calculations have been used to estimate the bond dissociation energies, which have been compared to experimental results of kinetic gas‐phase studies at high temperatures.^20^, ^21^, ^22^ So far, a computational study that includes the role of singlet oxygen in the reaction mechanism at a molecular level has not been performed.

Thus, the focus of this work was to investigate the reaction mechanism of the OER (related to the charging process) by employing a model system for which singlet oxygen generation can be considered in detail. Our starting point is the intermediately present lithium superoxide and the dissociation reaction towards the triplet/singlet oxygen molecule and lithium. In order to employ highly accurate multireference post‐Hartree–Fock methods such as CASSCF, complete active space perturbation theory of the second order (CASPT2), and multireference configuration interaction (MRCI), we chose molecular lithium superoxide as model system, since it contains just three atoms. The chosen methods are especially relevant for the description of excited states and accurate calculations of their dissociation processes.

Our goal was to identify whether the formation of singlet oxygen can take place from lithium superoxide on the molecular level. This goal was achieved by calculating the potential‐energy surfaces of different electronic states during the dissociation of lithium superoxide. Furthermore, we investigated whether the obtained dissociation behavior is similar for other alkali metal superoxides, namely, sodium superoxide and potassium superoxide. With the same question in mind, we considered similarities and differences between the alkali metal systems and the covalently bound system of the hydroperoxyl radical. Furthermore, we performed first test calculations for the LiO_2_ system including solvent effects of the electrolyte in order to qualitatively study their influence on the dissociation path. We believe that our tailored model system allows the fundamental underlying chemistry of the alkali metal/oxygen systems to be understood and that it can be used to make suggestions on how to avoid the formation of singlet oxygen molecules.

## Computational Details

All quantum chemical calculations were performed by using the software package MOLPRO v.2015.1.^23^, ^24^ The structure optimizations of the molecular superoxides HO_2_, LiO_2_, NaO_2_, and KO_2_ utilized the Hartree–Fock (HF) method, the multireference configuration interaction (MRCI) approach, the complete active space self‐consistent field (CASSCF) method,^25^, ^26^ and the second‐order complete active space perturbation theory (CASPT2)^24^ over the lowest doublet state. For the elements H, O, Li, and Na the Dunning‐type basis sets (aug)‐cc‐pVXZ (X=D,T,Q,5) were employed.^23^, ^27^, ^28^, ^29^, ^30^, ^31^ Basis‐set convergence was reached for (aug)‐cc‐pV5Z; thus, this basis set was applied for most of the calculations. The augmented functions have nearly no influence on the structure and energetics, so diffuse functions were just used in test calculations. These basis sets were not available for the element K; hence, we used the def2‐QZVPD basis set.^32^ In addition, we tested the def2‐QZVPD basis set for the element Na to test the comparability to the Dunning‐type basis set.

The point group of the hydroperoxyl radical (HO_2_) was determined to be *C_s_*, and the point group of the superoxides MO_2_ to be *C*_2*v*_ (M=Li, Na, K). The minimum structures were confirmed by frequency calculations at the CASSCF/CASPT2 level of theory. On the basis of the optimized structures, potential‐energy surfaces (PES) were calculated by using the MRCI, CASSCF, and CASPT2 methods and remaining symmetry. Thereby, we considered doublet and quartet spin states.

The CASSCF calculations employed the state average procedure (SA‐CASSCF), and all states were chosen to have equal weights. Different number of states were used and tested for the SA‐CASSCF calculations. The active space of LiO_2_ and NaO_2_ consisted of 13 electrons in 12 active orbitals [CASSCF(13,12)]; the orbitals are shown in Figure S1 of the Supporting Information for LiO_2_ as an example. The orbitals of the active space were chosen by considering different relevant points at the PES of the dissociation. The most important orbitals for the bonding nature and the description of the dissociation process of the alkali metal superoxides are the highest occupied $\pi_{\mathrm{OO}}^{*}$ orbitals of oxygen and the lowest unoccupied atomic orbital of the alkali metal atom 2s_Li_. Three virtual orbitals in the active space were Rydberg orbitals for a better description of correlations between metallic states and energetically high orbitals. These Rydberg orbitals contain higher molecular orbitals of oxygen and their linear combination with higher atomic orbitals of the metal atoms. A stable active space of KO_2_ was obtained by considering 13 electrons in 9 active orbitals [CASSCF(13,9)], whereby the three Rydberg orbitals 3π_OO_, 4π_OO_, 3$\pi_{\mathrm{OO}}^{*}$ are removed compared to LiO_2_/NaO_2_ because of a lower stability of the active space, probably due to the use of the non‐correlation‐consistent basis set. The active space of HO_2_ consisted of 13 electrons in 9 active orbitals [CASSCF(13,9)]. The orbitals of the active space are shown in Figure S2 of the Supporting Information. The orbitals of HO_2_ contain bonding contributions between oxygen orbitals and the 1s hydrogen orbitals. The same active spaces were selected for the MRCI calculations. Furthermore, the oxygen molecule in its triplet and singlet state as well as the superoxide anion O_2_^−^ were calculated at the CASSCF(12,8)/CASPT2 level of theory with a cc‐pV5Z basis set. The calculated bond lengths were determined to be 1.210 Å (^3^O_2_, ^3^∑_g_^−^), 1.220 Å (^1^O_2_,^1^Δ_g_), and 1.357 Å (O_2_^−^,^2^∏_g_).

A natural bond orbital (NBO) analysis with the NBO6.0 program was performed at the CASSCF level of theory to characterize the electronic states regarding their chemical bonding nature.^33^ The NBO method is based on the transformation of atomic basis functions to an orthonormal set of natural atomic orbitals (NAOs) with known occupation. The NAOs are used to form a set of polarized and hybrid coefficients, which are used to create the NBOs. In contrast to natural orbitals, NBOs are localized at one or two atoms. In the context of the NBO method the Wiberg bond index (WBI) was calculated. WBIs are the sum of the square nondiagonal matrix elements between atoms in the NAO representation. The analysis was employed for single states of different symmetry at the optimized ground‐state structures. For the KO_2_ system the bonding analysis was performed by using the restricted active space self‐consistent field method RASSCF(13,9) with the restriction of only considering the main configuration interaction (CI) state. The reason for this is a strong mixing of states by using the non‐correlation‐consistent basis set.

In order to study superoxide dissociation we constructed potential‐energy surfaces (PESs) of the molecular systems. The PES in two dimensions were calculated by considering four electronic states for the HO_2_ radical and eight electronic states for the MO_2_ radicals; for the PES in three dimensions we considered four electronic states. Two fundamental different two‐dimensional PES were generated: a PES created by linearly interpolated internal coordinates (LIIC), which we denote PES_LIIC_, and a PES with a fixed O−O bond length, which we call PES_fOO_.

For the PES_LIIC_ the starting point was chosen to be the superoxide energy minimum *R*_GS_. The final point was chosen to be a structure for which the energy states as well as the CI vectors no longer change along the reaction coordinates considering a O−O bond fixed with the O_2_ distance of triplet oxygen (1.210 Å). Thereby, a distance of about 4.000 Å for LiO_2_ and 4.500 Å for NaO_2_ between the alkali metal atom and the center of the oxygen dimer was reached, which is denoted *R*_Diss_.

Thus, the interpolation formula [Eq. 4]:

$$R_{i}\left(n+1\right)=R\left(n\right)+\frac{R_{Diss}-R_{GS}}{N}$$

with *n* as step number and *N* as total number of calculated points was employed. For PES_LIIC_ 52 points ranging from equilibrium distance *R*_GS_ to *R*_Diss_ were calculated for the different superoxides.

For the PES_fOO_ calculations the O−O bond lengths of the optimized superoxide structures were employed. Fixing the O−O bond had two reasons. First, the O−O distance variation during the dissociation (≈ 0.15 Å) does not change the qualitative picture of the dissociation pathway. Whereas the qualitative order of electronic states is unchanged, the energetics significantly changes. Thus, these PES_fOO_ were used to qualitatively interpret the dissociation paths. Second, accounting for a change in the O−O distance decreases the stability of the active spaces in many cases. This decrease in stability arises because higher‐order orbitals of the oxygen atoms participate in the active space during dissociation. Including all orbitals would lead to too large an active space, which cannot be treated. For the calculation of the HO_2_ radical only the PES_fOO_ was calculated. The PES_fOO_ for the HO_2_ radical dissociation was constructed by the variation of the H−O distance from 0.82 to 4.23 Å in 51 steps with a fixed bond angle *θ*_O‐O‐H_. Due to the higher symmetry of the MO_2_ superoxide systems, here the reaction coordinate was selected as distance between the metal atom and the center of the O−O bond. This distance was varied from 1.06 to 4.23 Å in 52 steps.

Furthermore, we constructed PESs to prove the obtained structures to be minima and to identify crossing points. The PESs of the alkali metal superoxides were constructed in a reduced symmetry, namely the *C_s_* point group. The bond lengths *R*_OO−Li_ and bond angles *θ*_O‐O‐M_ were varied, whereas the O−O bond distance was fixed. In addition, the PES along the bond lengths *R*_OO−M_ and *R*_O−O_ were calculated near determined crossing points. Here it was possible to include the change of the O−O coordinate due to the small crossing point area.

To include solvent effects in the dissociation process, test calculations with the conductor‐like screening model (COSMO) model were performed.^34^ These test calculations only have to be performed at the restricted open‐shell Hartree–Fock (ROHF) level of theory to consider the qualitative influence of solvent effects. The COSMO model is based on an electrostatic energy calculation of a system by applying screened charges. The charges are calculated from the electronic density and screened with a dielectric constant *f*(*ϵ*). Diglyme [bis(2‐methoxyethyl) ether] — a solvent commonly used for Na/O_2_ batteries — was accounted for in the COSMO model by using *ϵ*_r_=7.23.^35^

All molecular structures and orbitals were visualized with the program Chemcraft 1.8.^36^ Diagrams have been plotted with the programs Gnuplot 5.0, OriginPro 2016G, and Matlab R2018b.^37^, ^38^, ^39^

## Results and Discussion

### Determination of the molecular structures

**Figure 1:**
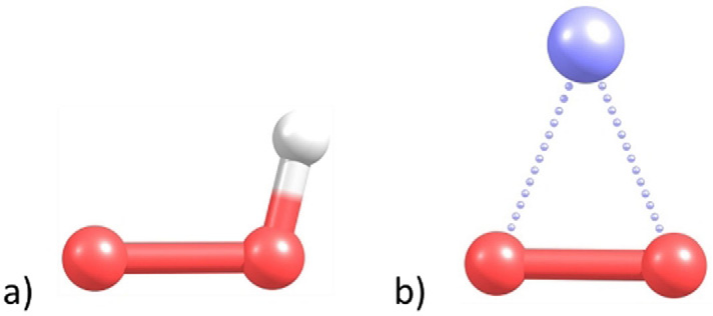
Minimum‐energy structures of the molecular superoxides. a) HO_2_ (*C_s_* point group) and b) MO_2_ (*C*_2*v*_ point group) for the example of LiO_2_ calculated at MRCI/aug‐cc‐pV5Z level of theory. Color code: O=red, H=white, Li=blue.

Structure optimizations of the superoxide systems were performed at the HF, CASSCF, CASSCF/CASPT2, and MRCI levels of theory. The CASSCF/CASPT2 results are summarized in Table 1, and the results of all other methods in Table S1 of the Supporting Information. Visualization of the two different fundamental structures is shown in Figure 1.

**Table 1:** Structural parameters of the calculated superoxides at the CASSCF/CASPT2/cc‐pV5Z level of for HO_2_, LiO_2_ and NaO_2_. For KO_2_ the Def2‐QZVPD basis set was applied.

System	Method	*R*_O−M(H)_ [Å]	*θ*_O‐M‐O(O‐O‐H)_ [°]	*R*_O−O_ [Å]
HO_2_	CASSCF(13,9)/CASPT2	0.973	104.32	1.331
	exptl^41^	0.977	104.1	1.335
LiO_2_	CASSCF(13,12)/CASPT2	1.766	45.08	1.353
	CASSCF(7,8)/TZP^22^	1.809	–	1.388
	exptl^40^	1.77±0.07	–	1.33±0.06
NaO_2_	CASSCF(13,12)/CASPT2	2.165	36.39	1.352
	exptl^42^	2.07	–	1.33±0.06
KO_2_	CASSCF(13,9)/CASPT2	2.480	31.72	1.355
	exptl^43^	2.10±0.14	37±2	1.33

The structure optimization of the molecular superoxides at the MRCI/aug‐cc‐pV5Z level of theory served as benchmark. The structures, which were optimized by the CASSCF(13,9/13,12)/CASPT2/aug‐cc‐pV5Z (K: def2‐QZVPD) methodology, agree very well with the MRCI benchmark. In particular, *R*_O−M_ and *R*_O−O_ bond lengths vary by less than 0.011 Å, and the *θ*_O‐M‐O_ and *θ*_O‐O‐H_ bond angles by less than 0.31°. The differences in structural parameters due to using the cc‐pV5Z basis set without diffuse basis functions compared to the aug‐cc‐pV5Z basis set are very small. Structures obtained by the CASSCF(13,9/13,12)/cc‐pV5Z approach also agree very well with the benchmark structures, whereas significant differences were obtained by just using the HF method.

The agreement of structural details of molecular LiO_2_ with the corresponding experimental results of refs. 22, 40 is reasonable, whereas there is a larger deviation from experimental data for the Na−O bond length of molecular NaO_2_. Also, our calculated K−O distance is much longer than the related experimental one. Considering the increasing ionic radii from lithium to potassium, we believe our calculated increase in M−O bond length to be reliable. The small experimental K−O distance may be due to a packing effect of the condensed KO_2_/Ar phase structure. This conclusion is supported by the comparison of the different MO_2_ molecules with solid‐state structures. Here, the increase of the M−O distance from LiO_2_ to NaO_2_ is similar to the increase from NaO_2_ to KO_2_. The comparison of the molecular alkali metal superoxide radicals with the solid‐state structures of the alkali metal superoxides is discussed in the Supporting Information (Table S2 and Figure S3).

The HO_2_ molecule with C_*s*_ point group exhibits a doublet ground (D_0_) state ^2^A′, and the alkali metal superoxides with *C*_2*v*_ point group have a doublet ground state ^2^A_2_. Calculations of the PESs while varying the M−O bond length and the *θ*_O‐M‐O_ and *θ*_O‐O‐H_ bond angles at CASSCF level of theory were carried out to confirm the minimum‐energy structures in addition to the frequency calculations. The corresponding Born–Oppenheimer surfaces of the ground state (D_0_) and the first excited state (D_1_) are plotted in Figure S4 of the Supporting Information. The superoxides HO_2_, NaO_2_ and KO_2_ exhibit only one stable structure in the ground state. For LiO_2_ a second possible structure with linear configuration but higher relative energy (about 0.75 eV) was identified. Corresponding potential‐energy curves of the angle dependence at a higher level of theory including dynamic correlation are plotted in Figure S5 of the Supporting Information. The molecular LiO_2_ structure previously calculated at the CASSCF(7,8)/TZP level of theory agrees well with our structure (see Table S1 of the Supporting Information).^22^ The linear configuration of LiO_2_ was also found by ROHF and CISD calculations in ref. 22.

### Analysis of the molecular structures and bonding nature

The structure, bonding nature, and the charge distribution of the hydroperoxyl radical (HO_2_) are different from those of the alkali metal superoxides. The HO_2_ molecule shows a slightly shorter O−O distance than the alkali metal superoxides and the calculated free superoxide anion (CASSCF(13,8)/CASPT2/cc‐pV5Z: 1.357 Å), which is in agreement with the experimental data.^39^ The WBI, which can be interpreted as the quantum chemical analogue to the bond order, together with the charge distribution indicates polar covalent binding for the angled HO_2_ molecule (see Table 2).

**Table 2:** Atomic charges and WBIs for the ground states of HO_2_ (H‐O1‐O2) and MO_2_ from NBO analysis at the CASSCF(13,12/13/9)/cc‐pV5Z (K: RASSCF(13,12)/Def2‐QZVPD) level of theory.

System	State	Charge [e]			WBI		
		H	O1	O2	O−O	H‐O1	H‐O2
HO_2_	D_0_ (A′′)	0.44	−0.43	−0.01	1.05	0.77	0.02

	State	M	O		O−O	M−O	
LiO_2_	D_0_ (A_2_)	0.92	−0.46		1.13	0.07	
NaO_2_	D_0_ (A_2_)	0.94	−0.47		1.14	0.04	
KO_2_	D_0_ (A_2_)	0.96	−0.48		1.09	0.03	

**Figure 2:**
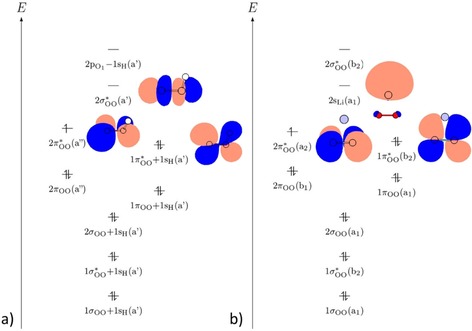
Schematic representation of the molecular orbital diagrams of the superoxides a) HO_2_ and b) MO_2_ (M=Li, Na, K), demonstrated by LiO_2_. In addition, the HOMO−1, HOMO, and LUMO are plotted.

This bonding nature is also supported by the molecular orbital diagram (see Figure 2 a and Figure S3 of the Supporting Information). There are σ_OH_ binding contributions in energetically low lying *σ*_OO_ and $\sigma_{\mathrm{OO}}^{*}$ molecular orbitals as well as in energetically higher lying π_OO_ and $\pi_{\mathrm{OO}}^{*}$ molecular orbitals.

All triangle‐shaped alkali superoxides MO_2_ (M=Li, Na, K) exhibit an O−O distance that agrees very well with the O−O distance of the free optimized superoxide anion. The *R*_O−M_ distances are strongly increased compared with HO_2_ (see Table 1). Interestingly, the *R*_O−M_ distance increases more from Li to Na (by about 0.39 Å) than from Na to K (by about 0.31 Å), whereas the ionic radii would indicate the opposite (increase of 0.26 Å from Li to Na and 0.36 Å from Na to K).^44^ In accordance with the increasing *R*_O−M_ distance, the bond angle *θ*_O‐M‐O_ decreases. For the metal superoxide radicals the WBI and charge distribution indicates an ionic bonding nature (see Table 2). This ionic character increases slightly from Li (0.92 e) to K (0.96 e), and is responsible for the higher‐symmetry molecular structure of the alkali metal superoxides without the presence of a localized bond as in HO_2_. However, the WBI indicates a small covalent binding contribution for LiO_2_. The molecular orbital diagram also indicates the ionic character (see Figure 2 b and Figure S4 of the Supporting Information). The *n*s_M_ orbital exhibits hardly any overlap with the oxygen orbitals (just slightly for LiO_2_). Thus, the molecular orbital diagram shows nearly an overlay of the orbital diagrams of the molecular superoxide anion and the atomic metal cation.

### Qualitative analysis of the dissociation processes

To evaluate and interpret the dissociation mechanism of superoxide, the calculated PESs of the molecular systems are used in the following. The PESs were constructed as two‐dimensional and three‐dimensional graphs of the energy for different electronic states over the main reaction coordinates for the dissociation.

#### Hydroperoxyl radical

**Figure 3:**
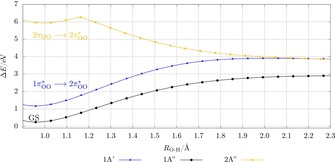
Dissociation curves of the three lowest electronic doublet states for HO_2_ with *θ*_O‐O‐H_=103.80° and *R*_O−O_=1.350 Å calculated at the CASSCF(13,9)/CASPT2/cc‐pV5Z level of theory. The character of the curves is given in the Franck–Condon region. For a better view, only three curves have been plotted.

The dissociation of the HO_2_ radical was calculated along the main reaction coordinate, the H−O distance, by PES_fOO_. Therefore, four states (two A*′* states, two A*′′* states) were considered. The corresponding atomic charges and WBIs of the two lowest states in the Franck–Condon region are given in Table S3 of the Supporting Information. The dissociation curves and the corresponding character of the states are shown in Figure 3. The ground‐state D_0_ curve exhibits no crossing with other states during the dissociation and represents the breaking of the localized covalent H−O bond to give a hydrogen atom and an oxygen molecule in its triplet state (^3^∑_g_^−^): HO_2_ → ^3^O_2_ +H. During this dissociation the molecular orbitals change to molecular orbitals with the shape of atomic orbitals (corresponding CI coefficients are given in Tables S5 and S6 of the Supporting Information). Our calculated ground‐state dissociation curve agrees well with the study on the reverse reaction of an H atom with O_2_, which shows no barrier.^45^ In addition, Walsh et al. investigated the conversion of the hydroperoxyl radical to OH and O, which we did not consider in the present work.^45^

The charge distribution and the WBI for the first excited state (1π^*^_OO_→2$\pi_{\mathrm{OO}}^{*}$ ) are very similar to those of the ground state and lie only about 1 eV higher in energy. The dissociation path along the first excited state (D_1_) leads to the generation of singlet oxygen (^1^Δ_g_). The second, D_2_, state exhibits an excitation from a 2π_OO_ orbital to a 2$\pi_{\mathrm{OO}}^{*}$ orbital, which also leads to generation of the singlet oxygen molecule (^1^Δ_g_).

#### Alkali metal superoxides

**Figure 4:**
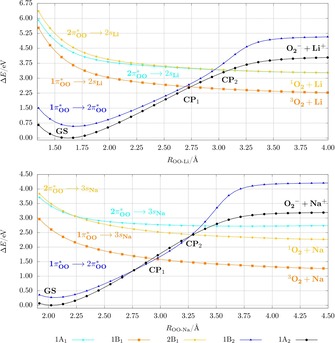
Dissociation curves with linear interpolated coordinates of the five energetically lowest electronic doublet states for LiO_2_ (top) and NaO_2_ (bottom) calculated at the CASSCF(13,12)/CASPT2/cc‐pV5Z level of theory (only five of the eight calculated states are shown for a better view). The character of the curves is given in the Franck–Condon region. The crossing points CP_1_ and CP_2_ are marked. The corresponding PES_fOO_ are plotted in Figure S6 of the Supporting Information and exhibit the same qualitative picture.

**Lithium superoxide**: The dissociation curves of the lithium superoxide model system were calculated by a PES_LIIC_ along the main reaction coordinate, which has been determined to be the distance between the lithium atom and the center of mass of the oxygen fragment. The eight lowest states corresponding to different symmetry in the Franck–Condon region (two A_1_ states, two A_2_ states, two B_1_ states, and two B_2_ states) were followed during the dissociation, although the focus was on the lowest four states. Next to the excitation to the 2π^*^_OO_ orbital — as for HO_2_ — especially the excitation to the 2s_Li_ orbital is present in the Franck–Condon region (see Figure 4 and Table S8 and the CI coefficients in Table S7 of the Supporting Information). The four energetically lowest states provide the ionic nature for A_2_ and B_2_ symmetry, and the uncharged van der Waals nature for A_1_ and B_1_ (see Table S4 of the Supporting Information).

The PES_LIIC_ of the dissociation and the most relevant characters of the states are plotted in Figure 4. The ground‐state curve D_0_ of the ionic bound lithium superoxide indicates a qualitatively different dissociation path compared with the covalent bound hydroperoxyl radical. The ground‐state curve crosses several other curves with different electronic character. This behavior indicates that electronically different dissociation products can be obtained by changing the pathway. Following the ground‐state D_0_ (1A_2_) curve, the dissociation path leads to the formation of Li^+^ and O_2_^−^, which in the gas phase is energetically less preferred than other paths.

The thermodynamically most stable dissociation product is reached following the first crossing point (CP_1_) with the Franck–Condon D_2_ state [π^*^_OO_ (b_2_) → 2s_Li_ (a_1_)], which leads to a triplet oxygen molecule (^3^∑_g_^−^) and a lithium atom for which the spin of the electron is in the opposite direction to those of triplet oxygen. A quartet state with all spins in the same direction and the same energy can be calculated as well (see Figure S7 of the Supporting Information). The results imply that the most preferable reaction path is the formation of the triplet oxygen molecule and lithium. The lithium atom might also be considered as the Li^+^/e^−^ pair but is not separated in our model. The reaction Li → Li^+^ + e^−^ would increase the potential energy in the dissociation limit. The crossing point indicates an electron‐transfer process at an Li−(OO) distance of about 2.75 Å. To reach the crossing point, an energy of about 2.5 eV corresponding to the PES_LIIC_ is necessary. Considering the reverse reaction of a lithium atom with triplet oxygen, this reaction can take place without or with just a small kinetic barrier.

**Figure 5:**
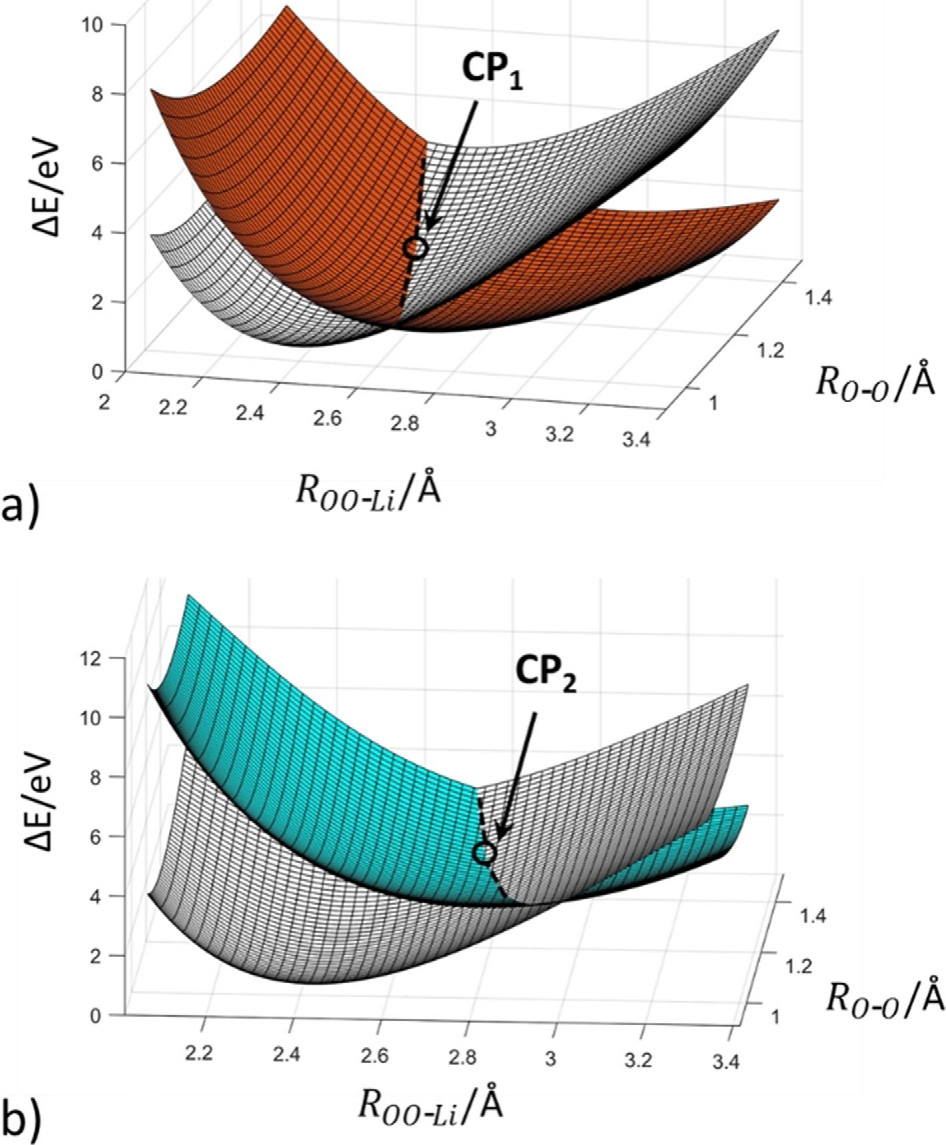
Born–Oppenheimer surfaces near the first and second minimum‐energy crossing points of doublet states for the LiO_2_ system calculated at the CASSCF(13,12)/cc‐pV5Z level of theory. The D_1_ state has been removed for a clearer view. Color code: D_0_ (1A_2_ black), D_2_ (1B_1_ orange), D_3_ (1A_1_ cyan).

The PES at the CASSCF/cc‐pV5Z level of theory near this first crossing point was calculated to determine the geometric structure including the variation of the O−O bond length (see Figure 5 and Figure S8 of the Supporting Information). It can be concluded that the O−O bond‐length change has a large influence on the energies but not on the other structural parameters and on the order of the different states. The PES (see Figure 5) shows a crossing seam mainly along the reaction coordinate *R*_O−O_ with the lowest energy difference between the states at values of *R*_OO−M_=2.62 Å and *R*_O−O_=1.26 Å. Thus, the PES_LIIC_ are able to qualitatively describe the dissociation paths in the relevant region. At crossing point CP_1_, the PES_LIIC_ (see Figure 4) and PES (see Figure S8) give evidence that nearly three states, that is, the two ionic states D_0_ and D_1_ as well as the nonionic state D_2_, are degenerate. The reason for the D_0_/D_1_ degeneration lies in the vanishing energy difference between the two $\pi_{\mathrm{OO}}^{*}$ (b_2_) energy levels along the dissociation path.

Following the D_0_ (1A_2_) curve even further leads to a second crossing point (CP_2_), which lies energetically about 0.9 eV higher than CP_1_. The second crossing point, which is characterized by the D_3_ and D_4_ states [1A_1_,1B_1_, $\pi_{\mathrm{OO}}^{*}$ (b_2_)→2s_Li_ (a_1_)], enables formation of a singlet oxygen molecule (^1^Δ_g_) and a lithium atom. The second crossing point indicates an alternative electron‐transfer step at an Li−(OO) distance of about 3.5 Å (PES‐determined structural parameters according to Figure 5 at the CASSCF level of theory: *R*_OO−Li_=2.91 Å, *R*_O−O_=1.23 Å). Also at this point, three states are nearly degenerate (see Figure S8 of the Supporting Information). For larger distances the degeneration of the two ionic states vanishes. There is nearly no additional kinetic barrier to overcome. The higher‐lying dissociation products were not considered in our study. Calculating the dissociation path of the linear LiO_2_ configuration results in the same qualitative picture, which implies that the general dissociation mechanism and the order of states do not change with the configuration of LiO_2_.

**Sodium and potassium superoxide**: We also calculated the PES_LIIC_ describing the dissociation of sodium superoxide (see Figure 4). For the KO_2_ system only the calculation of the PES_fOO_ with a long O−O distance was possible owing to instabilities of the active space (see Figure S9 of the Supporting Information). The order of the states and the character of the excitations in the Franck–Condon region and during the dissociation are the same as for the LiO_2_ system (the CI coefficients of the Franck–Condon region are listed in Tables S8 and S9 of the Supporting Information, and the WBIs and charge distributions in Table S4). Thus, curves qualitatively similar to those of LiO_2_ were obtained for the molecular systems NaO_2_ and KO_2_. For NaO_2_ only a slightly longer Na−(OO) distance for the first crossing point was found with a value of 2.95 Å. The energetically lowest path that leads to the formation of triplet oxygen lies about 1 eV lower for NaO_2_ and KO_2_ compared with LiO_2_. The energetic difference for the generation of triplet or singlet oxygen is between 0.81 and 0.89 eV higher depending on the alkali metal atom. The splitting of the states D_3_ and D_4_ for the PEC_LIIC_ of NaO_2_ occurs due to strong mixing of CI vectors of higher states. The D_3_ and D_4_ curves are degenerate on the PES_fOO_, which is physically more likely (see Figure S6 of the Supporting Information). In summary, the calculations demonstrate a similar dissociation mechanism for the ionically bound alkali metal superoxides, and thus, similar to the Li/O_2_ system, formation of singlet oxygen should be also possible for the Na/O_2_ and K/O_2_ systems.

#### Dissociation energies of the superoxide systems

The dissociation energies were calculated by considering the dissociation limit of the PES_fOO_ curves and correcting the energy dependent on O−O bond length to the equilibrium energy of triplet oxygen or singlet oxygen to get consistent values for all considered superoxides. In this way we obtained the ^3^ *E*_theo_ and ^1^ *E*_theo_ values (see Table 3). These values agree well with the values obtained from the PES_LIIC_ for LiO_2_ and NaO_2_. The ^3^ *E*_theo_ value decreases in the order H>Li>Na>K, whereas the dissociation energies for the set of HO_2_ and LiO_2_ and for the set of NaO_2_ and KO_2_, respectively, are almost equal. Including the thermodynamic correction and considering elevated temperatures of around 1000 K (as used in experimental studies; often flame/combustion studies) increases our calculated dissociation energies marginally by up to 0.1 eV. The large difference between the dissociation energies of LiO_2_ and the other alkali metal superoxides may be explained by the smaller ionic radius of lithium, associated with a stronger electrostatic interaction and a small covalent contribution of the Li−O bonds, which results in higher lying nonionic excited states. We also state ^1^ *E*_theo_ for the alkali metal superoxides, for which crossing points to singlet oxygen were observed on the PES. Here, the dissociation energies are about 0.8–0.9 eV higher than for the formation of the triplet oxygen molecule.

**Table 3:** Computed dissociation energies for the formation of triplet oxygen (^3^ *E*_theo_) and singlet oxygen (^1^ *E*_theo_) at the CASSCF(13,12)/CASPT2/cc‐pV5Z (K: CASSCF(13,9)/CASPT2/def2‐QZVPD) level of theory. The ^3^ *E*_theo_ values in parentheses include the thermodynamic correction at 298 K (the temperature of the kinetic experiments at 1100 K would lead to an additional energy change of 0.07 eV). All energies are given in electron volts.

Compound	^3^ *E*_theo_	^1^ *E*_theo_	Method	*E*_exptl_	Method	*E*_theo_
HO_2_	2.43	–	–		CASSCF/CI/ANO^45^	2.25
LiO_2_	2.24 (2.21)	3.14	gas‐phase study^20^	3.17	UMP4/6‐311G^20^	3.07
			flame study^46^	2.30	QZ2P+R+fUMP4(SDTQ)^22^:	2.56
					CCSD(T)/CBS‐DTQ^47^	2.59
NaO_2_	1.26 (1.30)	2.16	gas‐phase study^20^	2.09	UMP4/6‐311G^20^	1.92
			flame study^46^:	2.43	CCSD(T)/CBS‐DTQ^47^	1.58
			flame study^48^:	1.69		
			flame study^49^:	1.80		
KO_2_	1.04	1.83	CPFAAS^[a] [21]^	1.88	CCSD(T)/aug‐cc‐pwCV5Z^21^	1.85
			flame study^48^	1.80	CCSD(T)/CBS‐DTQ^47^	1.79
			flame study^49^	1.76		
			molecular‐beam study^50^	1.95

[a] Collinear photofragmentation and atomic absorption spectroscopy.

Experimental data for dissociation energies of the molecular LiO_2_ and NaO_2_ systems have been obtained from gas‐phase measurements by Plane et al. and Dougherty et al.^20^, ^46^ Dissociation energies of molecular KO_2_ have been reported in a number of studies, whereby gas‐phase measurements of Sorvajäri et al. represent the most recent work.^21^ Whereas the data for the experimentally determined dissociation energies of molecular LiO_2_ and NaO_2_ differ by 0.9 and 0.7 eV, respectively, the experimental values for KO_2_ agree well within 0.2 eV (see Table 3). For LiO_2_ and NaO_2_ the experimental dissociation energies are close to our calculated dissociation energies for the generation of triplet oxygen as well as for the generation of singlet oxygen. The experimental dissociation energies for KO_2_ are all close to our calculated case of singlet oxygen formation. However, the dissociation energies are usually determined by flame experiments, which are known to show large measurement errors.^21^ Thus, it could be possible that the experimental values are either overestimated or underestimated. Considering a possible overestimation, also the high dissociation energies for LiO_2_ and NaO_2_ as well as the experimental results of KO_2_ would agree with our calculated ^3^ *E*_theo_ value. In accordance with the experiments, the dissociation energies of molecular NaO_2_ and KO_2_ are quite close and about 1 eV lower in energy than that of LiO_2_. In summary, the comparison with experimental data indicates that the dissociation leads to the thermodynamically favored species, the triplet oxygen molecule. Since the experimental values reported vary and the formation of triplet oxygen is preferred on the calculated PES, it is likely that triplet oxygen was formed in the experiments we consider in our comparisons.

A comparison of our calculated multireference calculations to previous theoretical work is given in Table 3. The dissociation energy of HO_2_ calculated at the CASSCF level of theory by Walch et al. is slightly smaller than our value.^45^ Earlier theoretical investigations at the QCISD, UMP4, and CCSD(T) levels of theory for LiO_2_, NaO_2_ and KO_2_ yielded dissociation energies higher in energy than the values we obtained. The calculated dissociation energies by Allen et al., which are the most accurate ones related to the applied level of theory in the literature, as well as highly correlated single‐reference calculations using CCSD(T) for LiO_2_ and NaO_2_ are closer (difference of about 0.3 eV) to our calculated values than UMP4 results determined by Plane et al.^20^, ^22^, ^47^ The calculated CCSD(T) dissociation energies for KO_2_ are about 0.8 eV higher than our calculated ^3^ *E*_theo_ value and in agreement with the experimentally determined value. Our additional calculation of the KO_2_ system at the CCSD(T)/def2‐QZVPD level of theory resulted also in a formation energy for triplet oxygen of 1.95 eV. Thus, the difference of the ^3^ *E*_theo_ value calculated at the CASSCF(13,12)/CASPT2/def2‐QZVPD level of theory compared with the CCSD(T)/def2‐QZVPD value appears to be related to a larger multireference character of the system, not, for example, the basis set. A further investigation of the multireference character of the system might be necessary in order to understand the origin of this difference. In summary, the application of a more accurate level of theory, large active spaces for CASSCF, and large basis set allowed us to predict revised theoretical dissociation energies for LiO_2_, NaO_2_, and KO_2_ molecular systems.

#### Influence of the solvent on the molecular model systems

**Figure 6:**
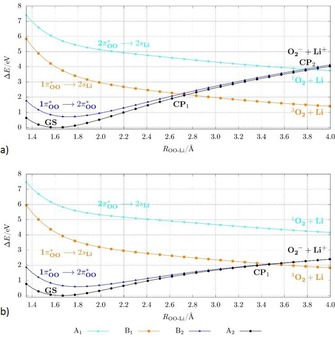
Dissociation curves of LiO_2_ calculated at ROHF/cc‐qV5Z level of theory a) in vacuum; and b) with solvent model of bis(2‐methoxyethyl) ether with *r*(Li)=2.57 Å (van der Waals), which is a common base component in the electrolyte of aprotic metal/oxygen batteries.

To estimate the impact of a solvent on the dissociation paths, we performed test calculations (only at the ROHF level of theory, Figure 6) on molecular LiO_2_ in diglyme, a solvent commonly used in the electrolyte of aprotic Na/O_2_ batteries.^51^ The obtained PES including solvent effects show a clear impact on the order of the electronic states of the curves. The application of a van der Waals radius for our applied solvent model results in a shift of the ionic curve between the curves for formation of triplet and singlet oxygen molecules. This would indicate a dissociation mechanism that leads either to triplet‐oxygen formation or to the superoxide anion and might be an advantageous way to avoid singlet‐oxygen formation, because this dissociation path is not reached. This shows that the dissociation mechanism of the molecular system can be significantly changed in solution by shifting of the electronic‐state curves. Also, a shift of the ground‐state curve below the curve that leads to generation of the oxygen molecule is possible. In this case a weakly interacting LiO_2_ ion pair would be formed, which has been already described in a combined study by DFT‐based molecular dynamics simulations and DFT on LiO_2_ in the explicit modeled solvents dimethyl sulfoxide, dimethoxyethane, and propylene carbonate.^52^ However, a more detailed study will be necessary to investigate the influence of the solvent effects at a higher level of theory as well as in combination with the structure of the metal oxide surface.

## Conclusion

To contribute to the fundamental understanding of the chemical processes during the charging of aprotic metal/oxygen batteries (focusing on lithium) and the corresponding decomposition of alkali metal superoxides, we considered the reaction leading from the intermediate lithium superoxide to the oxygen molecule and lithium. We studied the smallest possible model system, namely, molecular LiO_2_, in order to employ a high level of theory that allowed us to investigate different electronic states during the dissociation. Thus, we have performed high‐level CASSCF/CASPT2 calculations of the dissociation paths of the molecular structure of LiO_2_ to a triplet or singlet oxygen molecule and lithium. The dissociation of lithium superoxide leads via two different crossing points to the thermodynamically preferred triplet oxygen and a lithium atom or to the formation of singlet oxygen and a lithium atom via a path lying about 0.9 eV higher. Thus, the calculations clearly demonstrate two possible dissociation paths leading to a triplet or singlet oxygen molecule. This indicates the possibility of singlet‐oxygen formation for the molecular LiO_2_ system, but a higher driving force (e.g., higher voltage in the Li/O_2_ battery) than for the triplet‐oxygen formation is necessary. Depending on the probability of the electron jump from the initial superoxide ground state to the final curve of oxygen‐molecule formation, not only the energetically preferred path of the triplet oxygen molecule can be observed but also that of the singlet oxygen molecule. Our calculations give evidence that the peroxide molecule is not necessary as starting point for the generation of singlet oxygen. We note that the potential‐energy curves demonstrate fundamental differences between the ionically bound alkali metal superoxides and the covalently bound HO_2_.

Concerning the relevance of the results for metal/oxygen batteries, on the one hand the solvent effects of the environment need to be considered, and on the other hand the surface structure of the solid superoxides plays an integral role. Also, other battery components can influence the reaction. Furthermore, the application of an electric field must be implemented for future work. Regarding the electric field, we interpret our results by assuming a potential shift of stationary states. As for bulk or surface superoxides (experimental and theoretical studies), the gas‐phase reaction yields molecular oxygen. Anyhow, we clearly showed that the fundamental chemistry of alkali metal superoxide dissociation to different electronic states can be observed. The gained results indicate similar mechanisms of singlet‐oxygen formation for LiO_2_ and NaO_2_. Furthermore, formation may be also possible for KO_2_ systems by applying higher potentials and, at least for NaO_2_, there is already experimental evidence.^14^

**Figure 7:**
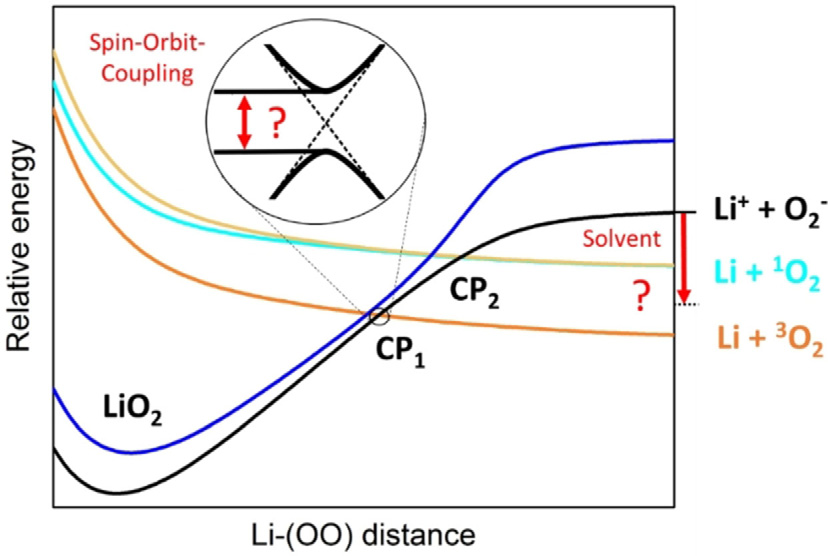
Sketch of possible manipulation points of the dissociation mechanism.

The gained knowledge on the underlying dissociation mechanism of the alkali metal superoxides can give access to strategies to avoid the formation of the highly reactive singlet oxygen. First, it could be investigated whether a beneficial choice of solvent/surface structure combination shifts the electronic dissociation curve with the path to the lithium cation and the superoxide anion between the formation curves for triplet and singlet oxygen, so that either triplet‐oxygen formation or dissociation to the ionic components takes place (see Figure 7). It is likely that this is also transferable to the disproportionation reaction, but this needs to be investigated in future research. Second, the manipulation of the crossing points by, for example, taking advantage of spin–orbit coupling effects by introducing heavy elements may be an option to avoid singlet‐oxygen formation by creating avoided crossings (see Figure 7). These suggestions go beyond the recently addressed use of quenching agents in the electrolyte of metal/oxygen batteries,^53^ which are aimed at deactivating the highly reactive singlet‐oxygen species during battery cycling. Our study can help to shape further research studies shedding light on how to prevent the formation of singlet oxygen in the first place, and ultimately help to minimize the deleterious influence of unwanted side reactions in aprotic metal/oxygen batteries.

## Conflict of interest

The authors declare no conflict of interest.

## Supporting information

As a service to our authors and readers, this journal provides supporting information supplied by the authors. Such materials are peer reviewed and may be re‐organized for online delivery, but are not copy‐edited or typeset. Technical support issues arising from supporting information (other than missing files) should be addressed to the authors.

Supplementary
