# Supplementary material for: Pathways to Triplet or Singlet Oxygen during the Dissociation of Alkali Metal Superoxides: Insights by Multireference Calculations of Molecular Model Systems
Source: Chemistry. 2020 Jan 21;26(11):2395–404. doi: 10.1002/chem.201904110 (PMC7187429; doi:10.1002/chem.201904110)
Supplement: Supplementary file 1 — Supplementary [file CHEM-26-2395-s001.pdf]

CHEMISTRY
A European Journal

Supporting Information

Pathways to Triplet or Singlet Oxygen during the Dissociation of Alkali Metal Superoxides: Insights by Multireference Calculations of Molecular Model Systems

Aleksandr Zaichenko, Daniel Schröder, Jürgen Janek, and Doreen Mollenhauer*[a]

chem_201904110_sm_miscellaneous_information.pdf

Author Contributions

A.Z. and D.M. designed the study. A.Z. performed the quantum chemical calculations. A.Z. and D.M. wrote the main part of the manuscript. All authors discussed the results and wrote contributions to the manuscript. All authors have given approval to the final version of the manuscript.

Information of the chosen active spaces

Figure S1: Orbitals of the active space of the LiO_2 molecular system calculated at the CASSCF(13,12)/cc-pV5Z level of theory and labeled with symmetry and character (the three orbitals at the bottom are Rydberg orbitals).

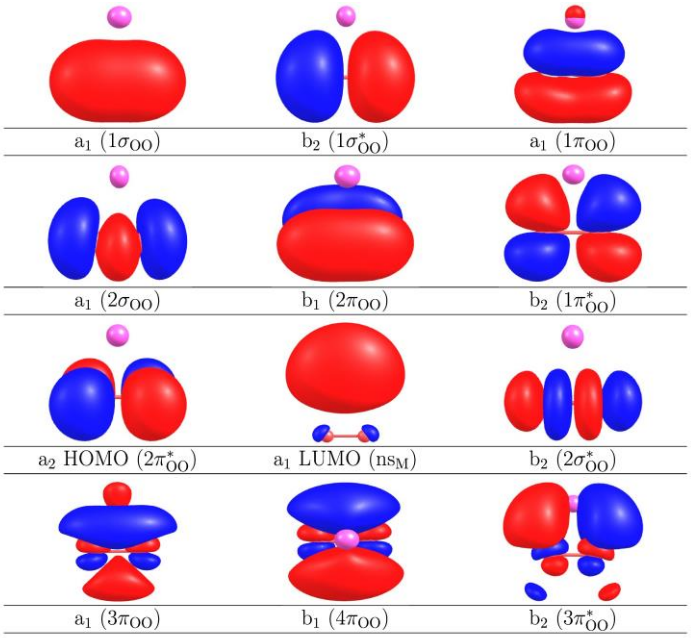

Figure S2: Orbitals of the active space of the HO_2 molecule calculated at the CASSCF(13,9)/cc-pV5Z level of theory and labeled with symmetry and character.

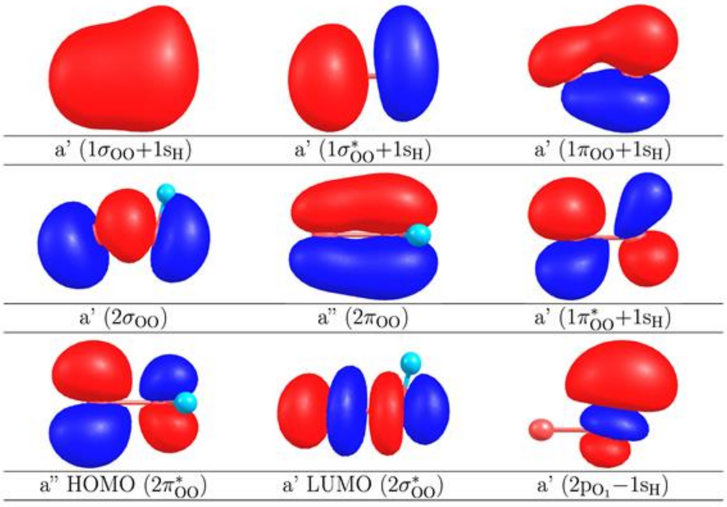

Structural parameters of optimization

Table S1 Structural parameters of the superoxides calculated at different level of theory. The ROHF and CASSCF/CASPT2 calculations have been performed using the cc-pV5Z basis set, the MRCI calculations using aug-cc-pV5Z for HO_2, LiO_2 and NaO_2. For KO_2 the Def2-QZVPD basis set was applied for all calculations.

System	Method	$R_{\text{O-M(O-H)}}$/Å	$\theta_{\text{O-O-M(H)}}$/°	$R_{\text{O-O}}$/Å
HO_2	RHF	0.945	106.15	1.302
	CASSCF(13,9)	0.974	103.29	1.349
	CASSCF(13,9)/CASPT2	0.973	104.32	1.331
	MRCI(13,9)	0.969	104.06	1.332
	Exp.[1]	0.977	104.1	1.335
LiO_2	RHF	1.729	43.47	1.281
	CASSCF(13,12)	1.766	45.40	1.363
	CASSCF(13,12)/CASPT2	1.766	45.08	1.353
	MRCI(13,12)	1.763	45.13	1.353
	CASSCF(7,8)/TZP[2]	1.809	-	1.388
	Exp. [3]	1.77±0.07	-	1.33±0.06
NaO_2	RHF	2.116	35.28	1.283
	CASSCF(13,12)	2.159	36.92	1.367
	CASSCF(13,12)/CASPT2	2.165	36.39	1.352
	MRCI(13,12)	2.155	36.69	1.356
	Exp.[4]	2.07	-	1.33±0.06
KO_2	RHF	2.437	30.42	1.279
	CASSCF(13,9)/CASPT2	2.480	31.72	1.355
	MRCI(13,9)	2.469	31.77	1.352
	Exp. [5]	2.10±0.14	37±2	1.33

Comparison of the calculated molecular radicals to bulk phases

Table S2: Comparison of interatomic distances (the minimal value is shown) of experimental determined orthorhombic LiO_2, NaO_2 and tetragonal KO_2 solid state structures with calculated molecular structures at CASSCF(13,12)/CASPT2/cc-pV5Z and CASSCF(13,9)/CASPT2 def2-QZVPD level of theory.

	Experimental solid state structures [6-8]		Calculated molecular structures	
	$R_{O\text{-}M}$/Å	$R_{O\text{-}O}$/Å	$R_{O\text{-}M}$/Å	$R_{O\text{-}O}$/Å
LiO_2	2.095	1.344	1.776	1.353
NaO_2	2.394	1.283	2.165	1.352
KO_2	2.710	1.280	2.480	1.355

The comparison of the molecular LiO_2 radical to the orthorhombic bulk structure (space group Pnnm) exhibits a good agreement of the O-O bond distance (1.344 Å) whereas the $R_{O\text{-}M}$ bond length is much smaller due to the different environment. The large $R_{O\text{-}M}$ bond distance in bulk crystals is also confirmed by theoretical modelling results [9]. In the bulk phase the superoxide radical is surrounded by six lithium atoms whereas each oxygen shows a distance of about 2.1 Å to three lithium atoms and about 2.9 Å to the other three atoms (see Figure S3). In another LiO_2 solid state structure (space group Pnnm) of Farley *et al.* the superoxide with a bond length of more than about 2.3 Å appears to be dissociated, thus, we have not considered this bulk phase in the Table S2 [6]. The solid state structure of NaO_2 (space group Pnnm) reveals a smaller O-O bond distance (1.283 Å) than the molecular system. The $R_{O\text{-}M}$ distances are increased to about 2.4 Å and 3.2 Å relatively similar to the LiO_2 system.[7] Also, the tetragonal bulk phase of KO_2 (space group I4/mmm) exhibits a similar small O-O bond distance (1.280 Å) as bulk NaO_2 and increased $R_{O\text{-}M}$ distances of about 2.7 Å and 2.9 Å. [8] However, the monoclinic solid state of

KO_2 (space group C12/c1) shows unrealistic small $R_{O\text{-}O}$ and $R_{O\text{-}M}$ distances, so that we have excluded this bulk phase from our comparison [10].

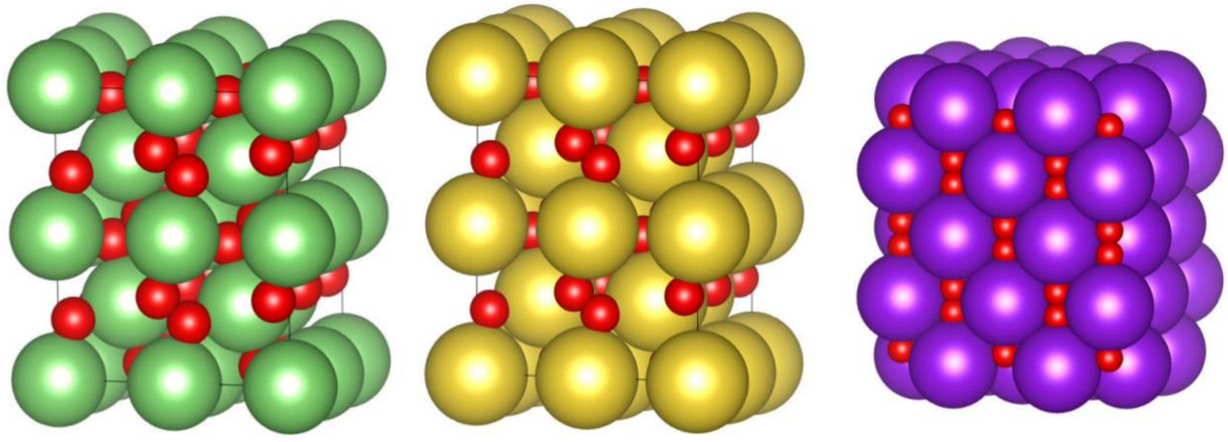

a) b) c)

Figure S3: Bulk structures of alkali metal superoxides a) LiO_2 b) NaO_2 c) KO_2.

Additional potential energy surfaces

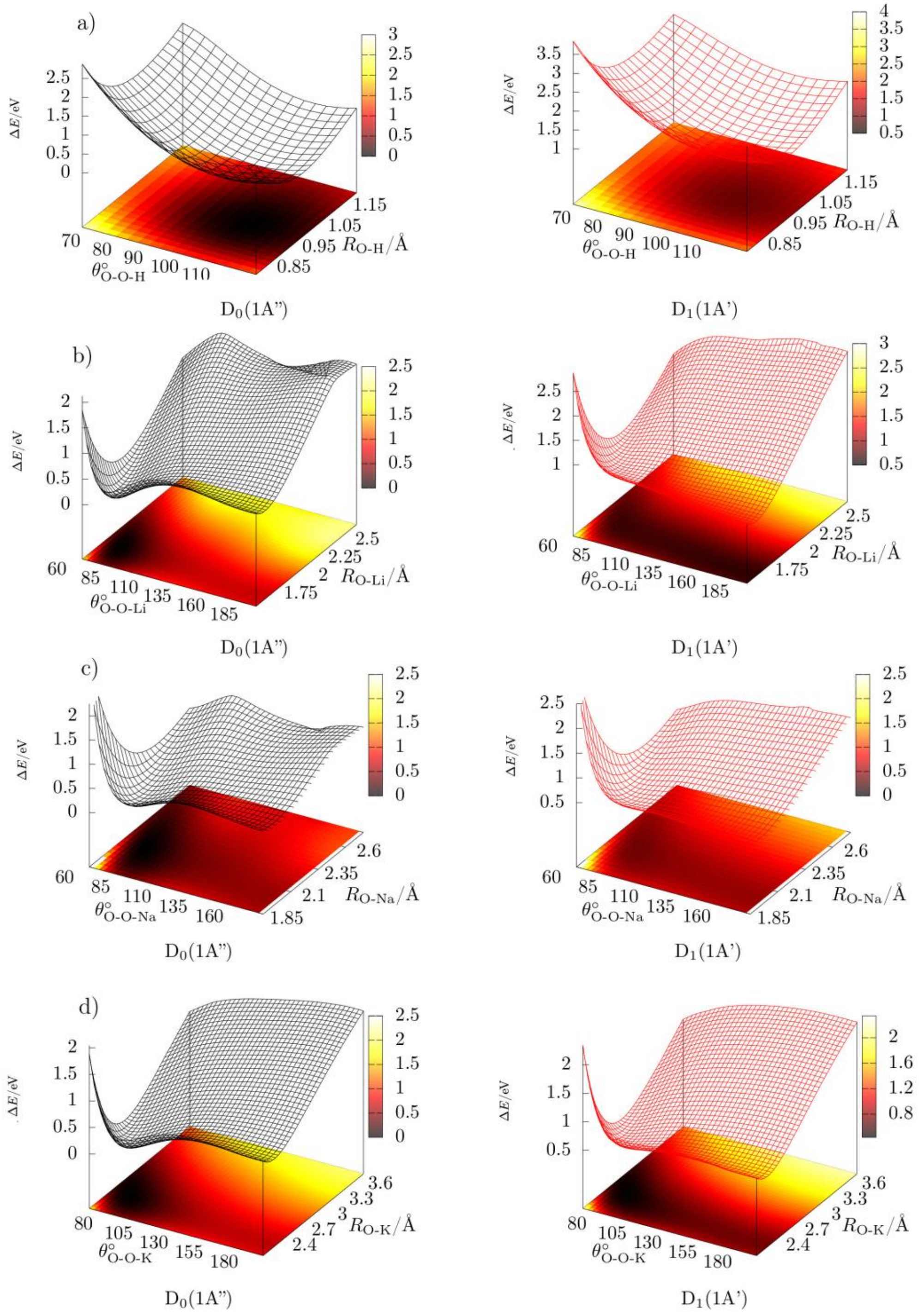

Figure S4: Potential energy surfaces of the molecular superoxides varying the $R_{O\text{-}X}$ bond lengths and the $\theta_{O\text{-}O\text{-}X}$ bond angle for a) HO_2 calculated at CASSCF(13,9)/cc-pV5Z level with $R_{O\text{-}O}$ =1.350 Å; b) LiO_2 calculated at CASSCF(13,12)/cc-pV5Z level with $R_{O\text{-}O}$ =1.350 Å; c) NaO_2 calculated at CASSCF(13,12)/cc-pV5Z level with $R_{O\text{-}O}$ =1.350 Å and d) KO_2 calculated at CASSCF(13,9)/def2-QZVPD level with $R_{O\text{-}O}$ =1.430 Å.

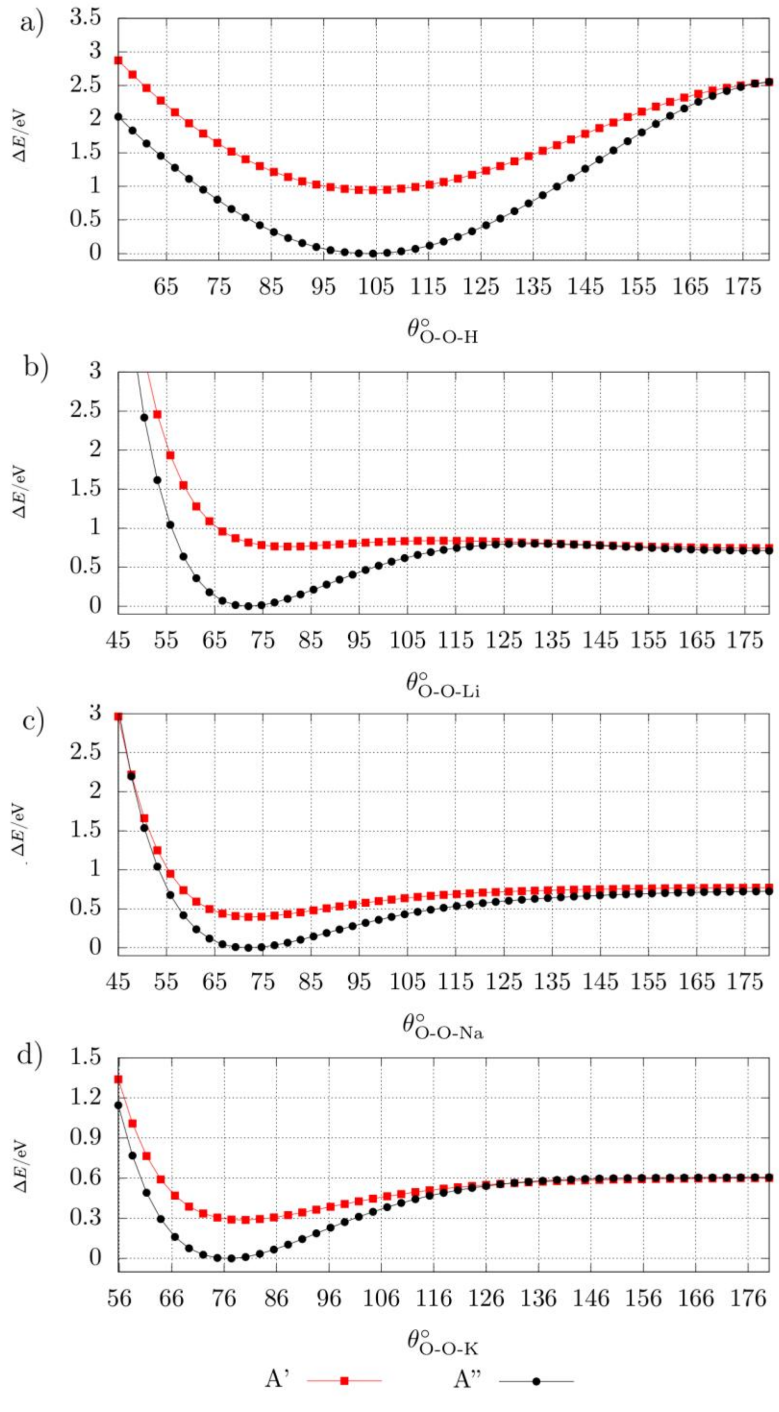

Figure S5: Potential energy surfaces of the molecular superoxides varying the $\theta_{O\text{-}O\text{-}X}$ bond angle for a) HO_2 calculated at CASSCF(13,9)/CASPT2/cc-pV5Z level with $R_{O\text{-}O}$ =1.350 Å and $R_{O\text{-}H}$ =0.951 Å; b) LiO_2 calculated at CASSCF(13,12)/CASPT2/cc-pV5Z level with $R_{O\text{-}O}$ =1.350 Å and $R_{O\text{-}Li}$ =1.61 Å; c) NaO_2 calculated at CASSCF(13,12)/CASPT2/cc-pV5Z level with $R_{O\text{-}O}$ = 1.350 Å and $R_{O\text{-}Na}$ = 2.170 Å; d) KO_2 calculated at CASSCF(*13,9*) /CASPT2/def2-QZVPD level with $R_{O\text{-}O}$ = 1.430 Å and $R_{O\text{-}K}$ = 2.380 Å.

Additional atomic charges and Wiberg bond indexes of excited states

Table S3: Atomic charges (e) and Wiberg bond indexes (WBI) for the first two states of HO_2 (H-O1-O2) from NBO analysis at CASSCF(13,9)/cc-pV5Z theory level. Oxygen O1 bonds with hydrogen and second oxygen O2.

State	Charge (e)			WBI		
	H	O1	O2	O1-H	O1-O2	H-O2
D_0 (A'')	0.44	-0.43	-0.01	0.766	1.047	0.016
D_1 (A')	0.42	-0.32	-0.10	0.746	0.972	0.006

Table S4: Atomic charges (e) and Wiberg bond indexes (WBI) of the minimum structure for the first four states of MO_2 from NBO analysis at CASSCF(13,12/13/9)/cc-pV5Z (K: RASSCF(13,12)/Def2-QZVPD) theory level.

State	LiO_2				NaO_2				KO_2			
	Charge (e)		WBI		Charge (e)		WBI		Charge (e)		WBI	
	Li	O	Li-O	O-O	Na	O	Na-O	O-O	K	O	K-O	O-O
D_0 (A_2)	0.92	-0.46	0.068	1.132	0.94	-0.47	0.042	1.136	0.96	-0.48	0.025	1.090
D_1 (B_2)	0.94	-0.47	0.043	1.125	0.96	-0.48	0.032	1.122	0.96	-0.48	0.027	1.248
D_2 (B_1)	0.00	0.00	0.034	1.290	0.00	0.00	0.022	1.288	0.03	-0.02	0.028	1.504
D_3 (A_1)	0.00	0.00	0.036	1.559	0.00	0.00	0.023	1.549	0.02	-0.01	0.026	2.007

Configuration state vectors at important structural points

Table S5: Configuration interaction (CI) state vectors of the lowest HO_2 states of the minimum structure calculated at CASSCF(13,9)/cc-pV5Z level of theory with coefficients $c > 0.1$.

Orbitals in the active space with irreducible representation		
**a‘**<br>$1\sigma^*_{OO}$ $1\sigma_{OO}$ $1\pi_{OO}$ $2\sigma_{OO}$ $1\pi^*_{OO}$ $2\sigma^*_{OO}$ $1\sigma_{OH}$	**a‘‘**<br>$2\pi_{OO}$ $2\pi^*_{OO}$	
Occupation of the orbitals		CI coefficients
**State D_0(A”)**		
2 2 2 2 2 0 0	2 α	0.97
2 2 2 0 2 2 0	2 α	-0.12
2 2 2 β 2 α 0	α 2	0.10
**State D_1(A’)**		
2 2 2 2 α 0 0	2 2	0.98
2 2 2 0 α 2 0	2 2	-0.14

Table S6: Configuration interaction (CI) state vectors of the lowest HO_2 states of the dissociated system (at $R_{O\text{-}H}$ = 2.300 Å) calculated at CASSCF(13,9)/cc-pV5Z level of theory with coefficients c > 0.1.

Orbitals in the active space with irreducible representation		
**a'** $1\sigma^*_{OO}1\sigma_{OO}1\pi_{OO}2\sigma_{OO}1\pi^*_{OO}2\sigma^*_{OO}1\sigma_{OH}$	**a''** $2\pi_{OO}2\pi^*_{OO}$	
Occupation of the orbitals		CI coefficients
**State D_1(A')**		
2 2 2 2 0 α 0	2 2	0.663
2 2 2 2 2 α 0	2 0	-0.663
**State D_0(A")**		
2 2 2 2 α β 0	2 α	0.777
2 2 2 2 α α 0	2 β	-0.389
2 2 2 2 β α 0	2 α	-0.389

Table S7: Configuration interaction (CI) state vectors of lowest LiO_2 states of different symmetry of the minimum structure calculated at CASSCF(13,12)/cc-pV5Z level of theory with coefficients $c > 0.1$.

Orbitals in the active space with irreducible representation				
**a_1**<br>$1\sigma_{OO}1\pi_{OO}2\sigma_{OO}$ $2s_{Li}3\pi_{OO}$	**b_1**<br>$2\pi_{OO}4\pi_{OO}$	**b_2**<br>$1\sigma^*_{OO}1\pi^*_{OO}2\sigma^*_{OO}3\pi^*_{OO}$	**a_2**<br>$2\pi^*_{OO}$	
Occupation of the orbitals				CI coefficients
**State $D_0(A_2)$**				
2 2 2 0 0	2 0	2 2 0 0	α	0.957
2 2 β 0 0	α 0	2 2 α 0	2	0.108
2 2 0 0 0	2 0	2 2 2 0	α	-0.108
**State $D_1(B_2)$**				
2 2 2 0 0	2 0	2 α 00	2	0.960
2 α β 0 0	2 0	2 2 α 0	2	-0.106
2 2 0 0 0	2 0	2 α 2 0	2	0.106
**State $D_2(B_1)$**				
2 2 2 β 0	2 0	2 α 0 0 α	α	0.769
2 2 2 α 0	2 0	2 α 0 0	β	-0.401
2 2 2 α 0	2 0	2 β 0 0	α	-0.369
2 α 2 β 0	α 0	2 2 0 0	2	0.124
**State $D_3(A_1)$**				
2 2 2 α 0	2 0	2 2 0 0	0	0.908
2 2 2 α 0	0 0	2 2 0 0	2	-0.259

2 2 2 α 0	2 0	2 0 0 0	2	-0.172
2 2 0 α 0	2 0	2 2 2 0	0	-0.105

Excitations of the molecular LiO_2

Table S7: Excitation character of the different electronic states of LiO_2 at the minimum structure and dissociated structure.

State	Franck-Condon region	Close to dissociated systems
D_0 ($1A_2$)	Ground state (lithium cation and superoxide anion)	lithium cation and superoxide anion
D_1 ($1B_2$)	$1\pi^*_{OO} \rightarrow 2\pi^*_{OO}$	lithium cation and superoxide anion
D_2 ($1B_1$)	$1\pi^*_{OO} \rightarrow 2s_{Li}$	lithium atom and $^3\Sigma_g^-$ triplet oxygen
D_3 ($1A_1$)	$2\pi^*_{OO} \rightarrow 2s_{Li}$	lithium atom and $^1\Delta_g$ singlet oxygen
D_4 ($1B_1$)	$1\pi^*_{OO} \rightarrow 2s_{Li}$<br>$1\pi^*_{OO} \rightarrow 2\pi^*_{OO}$	lithium atom and $^1\Delta_g$ singlet oxygen
D_5 ($2A_1$)	$1\pi^*_{OO} \rightarrow 2s_{Li}$	lithium atom and $^1\Sigma_g^+$ singlet oxygen

Additional PES of the dissociation

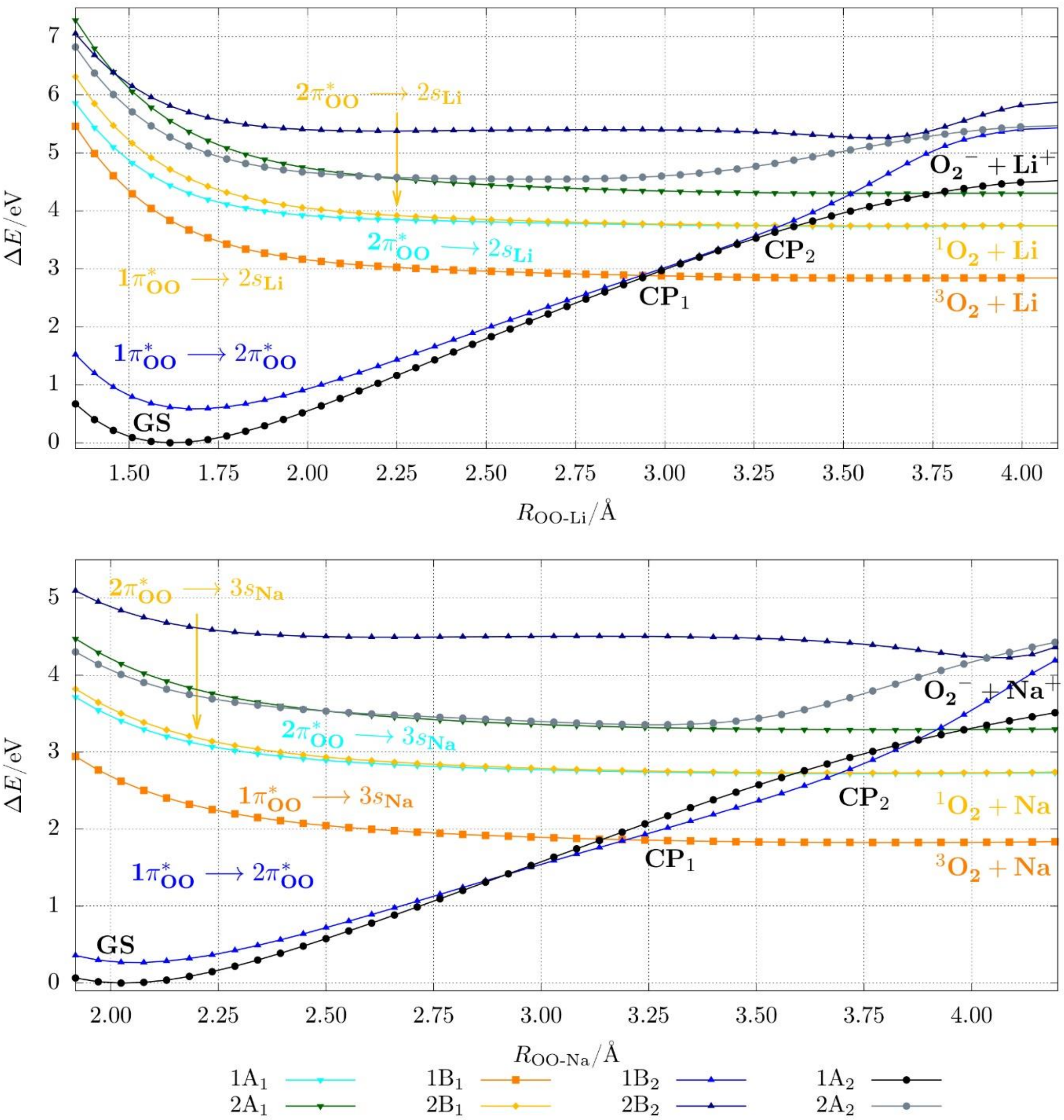

Figure S6: Dissociation curves / PEC_{fOO} of the eight lowest electronic doublet states for LiO_2 (upper picture) and NaO_2 (lower picture) calculated at CASSCF(13,12)/CASPT2 cc-pV5Z level of theory with fixed $R_{O\text{-}O}$ value (1.350 Å).

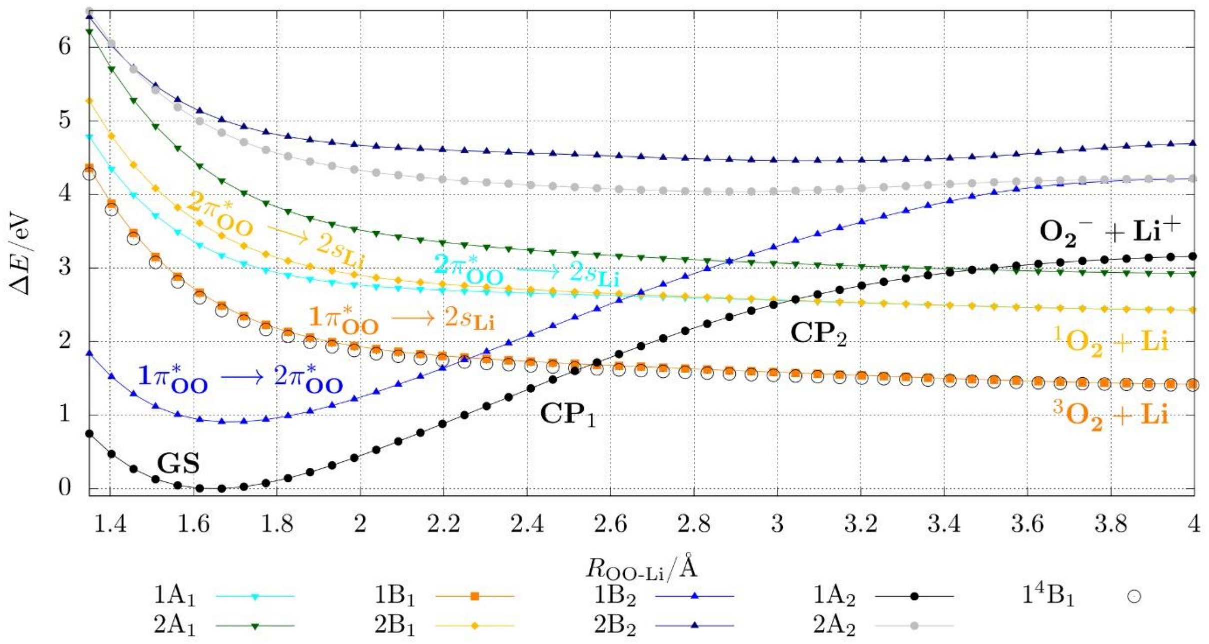

Figure S7: Dissociation curves of the eight lowest electronic doublet states including one quartet state for LiO_2 calculated at CASSCF(13,12/ cc-pV5Z level of theory with fixed $R_{O\text{-}O}$ value (1.350 Å).

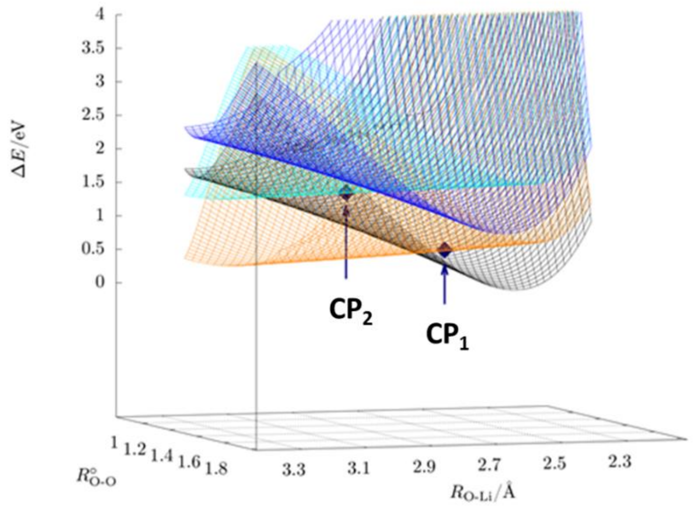

Figure S8: Born-Oppenheimer surfaces near the first and second crossing point of doublet states for LiO_2 calculated at CASSCF(13,12)/cc-pV5Z level of theory. Color code: D_0 ($1A_2$ black), D_1 ($1B_2$ blue), D_2 ($1B_1$ cyan), D_3 ($1A_1$ orange).

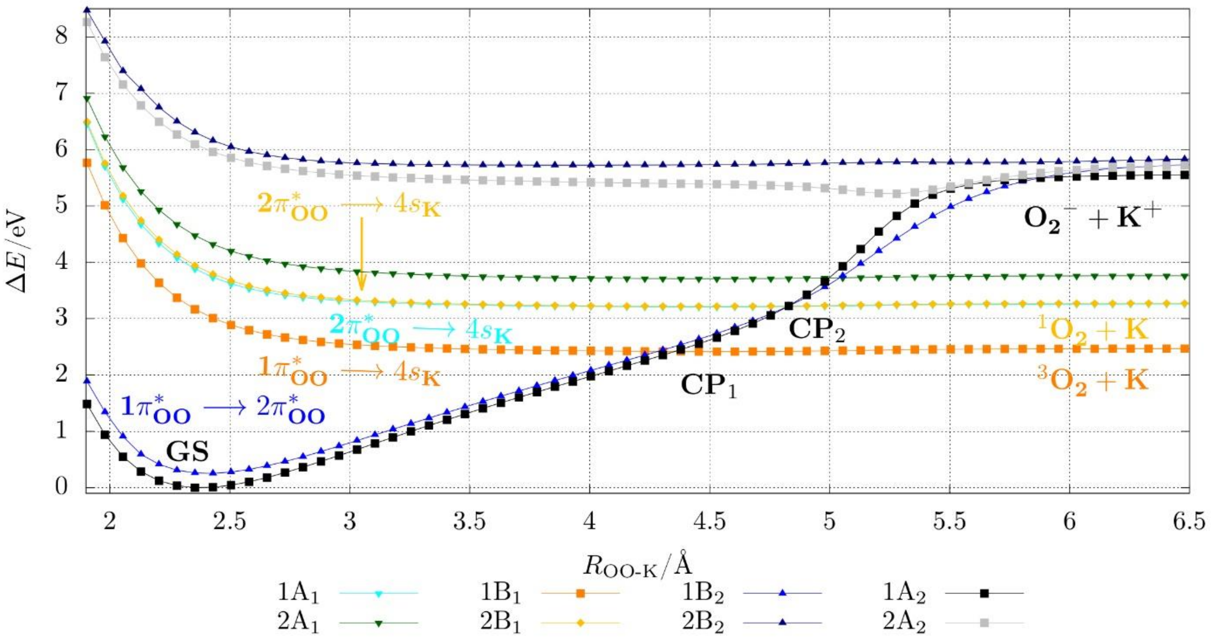

Figure S9: Dissociation curves of the eight lowest electronic doublet states of KO_2 with fixed $R_{O\text{-}O}$ = 1.430 Å calculated at CASSCF(13,9)/CASPT2/def2-QZVPD level of theory.

Additional data to describe the minima structures and relevant points of the PES

Table S8: Configuration interaction (CI) state vectors of lowest NaO_2 states of different symmetry of the minimum structure calculated at CASSCF(13,12)/cc-pV5Z level of theory with coefficients $c > 0.1$.

Orbitals in the active space with irreducible representation				
**a_1**<br>$1\sigma_{OO}1\pi_{OO}2\sigma_{OO}ns_M3\pi_{OO}$	**b_1**<br>$2\pi_{OO}4\pi_{OO}$	**b_2**<br>$1\sigma^*_{OO}1\pi^*_{OO}2\sigma^*_{OO}3\pi^*_{OO}$	**a_2**<br>$2\pi^*_{OO}$	
Occupation of the orbitals				CI coefficients
**State $D_0(A_2)$**				
2 2 2 0 0	2 0	2 2 0 0	α	0.958

2 2 β 0 0	A 0	2 2 α 0	2	0.104
**State $D_1(B_2)$**				
2 2 2 0 0	2 0	2 α 0 0	2	0.958
2 α β 0 0	2 0	2 2 α 0	2	-0.102
**State $D_2(B_1)$**				
2 2 2 β 0	2 0	2 α 0 0	α	0.768
2 2 2 α 0	2 0	2 α 0 0	β	-0.394
2 2 2 α 0	2 0	2 β 0 0	α	-0.373
2 α 2 β 0	A 0	2 2 0 0	2	0.127
**State $D_3(A_1)$**				
2 2 2 α 0	2 0	2 2 0 0	0	0.888
2 2 2 α 0	0 0	2 2 0 0	2	-0.255
2 2 2 α 0	2 0	2 0 0 0	2	-0.255

Table S9: Configuration interaction (CI) state vectors of lowest KO_2 states of different symmetry of the minimum structure calculated at CASSCF(13,12)/cc-pV5Z level of theory with coefficients $c > 0.1$.

Orbitals in the active space with irreducible representation				
**a_1** $1\sigma_{OO}1\pi_{OO}2\sigma_{OO}$ ns_M	**b_1** $2\pi_{OO}$	**b_2** $1\sigma^*_{OO}1\pi^*_{OO}2\sigma^*_{OO}$	**a_2** $2\pi^*_{OO}$	
Occupation of the orbitals				CI coefficients
**State $D_0(A_2)$**				
2 2 2 0	2	2 2 0	α	0.964
2 β 2 0	α	2 2 α	2	0.153

2 0 2 0	2	2 2 2	α	-0.129
2 α 2 0	α	2 2 β	2	-0.124
**State $D_1(B_2)$**				
2 2 2 0	2	2 α 0	2	0.963
2 β α 0	2	2 2 α	2	0.153
2 0 2 0	2	2 α 2	2	-0.129
2 α α 0	2	2 2 β	2	-0.124
**State $D_2(B_1)$**				
2 2 2 β	2	2 α 0	α	0.766
2 2 2 α	2	2 α 0	b	-0.382
2 2 2 α	2	2 β 0	α	-0.384
2 2 α β	α	2 2 0	2	0.172
2 0 2 β	2	2 α 2	α	-0.101
**State $D_3(A_1)$**				
2 2 2 α	2	2 2 0	0	0.860
2 2 2 α	2	2 0 0	2	-0.320
2 2 2 α	0	2 2 0	2	-0.290
2 0 2 α	2	2 2 2	0	-0.127

REFERENCES

[1] Y. Beers and C. J. Howard, J. Chem. Phys. 64(4), 1541-1543 (1976).

[2] W. D. Allen, D. A. Horner, R. L. Dekock, R. B. Remington, and H. F. Schaefer, Chem. Phys. 133(1), 11-45 (1989).

[3] L. Andrews, J. Chem. Phys. 50(10), 4288-4299 (1969).

[4] L. Andrews, J. Chem. Phys. 73(11), 3922-3928 (1969).

[5] B. Tremblay, L. Manceron, P. Roy, A.-M. Le Quéré, and D. Roy, Chem. Phys. Lett. 228(4-5), 410-416 (1994).

[6] T. W. D. Farley, W. Hayes, S. Hull, M. T. Hutchings, M. Vrtis, J. Phys. Condens. Matter, 3(26), 4761-4781 (1991).

[7] G. F. Carter, D. H. Templeton, J. Am. Chem. Soc. 75(21), 5247-5249 (1953).

[8] S. C. Abrahams, J. Kalnajs, Acta Cryst. 8(8), 503-506 (1955).

[9] Y. N. Zhuravlev, O. S. Obolonskaya, J. Struct. Chem. 51, 1005-1013 (2010).

[10] M. Ziegler, M. Rosenfeld, W. Känzig and P. Fischer, Helv. Phys. Acta. 49, 57–90 (1976).
